# Local infiltration analgesia versus peripheral nerve block anaesthesia in total knee arthroplasty: a pharmaco-economic comparison

**DOI:** 10.1186/s12871-022-01620-w

**Published:** 2022-03-25

**Authors:** Michael Borck, Jan D. Wandrey, Moritz Höft, Joanna Kastelik, Carsten Perka, Sascha Tafelski, Sascha Treskatsch

**Affiliations:** 1 Charité – Universitätsmedizin Berlin, Corporate Member of Freie Universität Berlin, Humboldt-Universität zu Berlin, Charitéplatz 1, 10117 Berlin, Germany; 2 Berlin Institute of Health , Charitéplatz 1, 10117 Berlin, Germany; 3 Department of Anaesthesiology and Intensive Care, Campus Charité Mitte and Campus Virchow-Klinikum, Augustenburger Platz 1, 13353 Berlin, Germany; 4 Centre for Musculoskeletal Surgery, Campus Charité Mitte, Charitéplatz 1, 10117 Berlin, Germany; 5 Department of Anaesthesiology and Intensive Care, Charité Campus Benjamin Franklin (CBF), Hindenburgdamm 30, 12203 Berlin, Germany

**Keywords:** Local infiltration analgesia, Peripheral nerve block, Pain management, Cost comparison

## Abstract

**Background:**

A superior analgesic method in perioperative pain-management of patients receiving total knee arthroplasty is the subject of controversial debate. Although higher cost-efficiency is claimed for the local infiltration analgesia (LIA), there is a lack of data on its costs compared to peripheral nerve block anaesthesia (PNBA). The goal of this study was to investigate the differences in immediate perioperative costs between the LIA and PNBA in treatment of patients receiving total knee arthroplasty.

**Methods:**

The comparison was conducted based on a randomized controlled clinical trial examining 40 patients with elective, primary total knee arthroplasty (TKA, 20 patients with LIA and 20 patients with PNBA). The analysis included surgical case costs, anaesthesiological case costs, material, costs of postoperative opioid requirements and catheter review visits for patients receiving PNBA.

**Results:**

The overall mean costs for the LIA-group were 4328.72€ and 4368.12€ for the PNBA (*p* = 0.851). While there was no statistically significant difference in surgical case costs, the anaesthesiological costs were lower with the LIA procedure (1370.26€ vs. 1542.45€, *p* = 0.048). Material costs in the LIA group were 4.18€/patient and 94.64€/patient with the PNBA. Costs for postoperative opioid requirements showed no statistically significant difference between the two procedures.

**Conclusions:**

There is no relevant difference in immediate perioperative costs between LIA and PNBA. Shorter induction times lead to lower anaesthesiological case costs with the LIA. Overall economic aspects seem to play a less important role for determining an adequate procedure for perioperative pain management.

**Trial registration:**

The study was approved by the ethics-review-board of Charité Hospital Berlin (Ethikausschuss 4, Charité – Universitätsmedizin Berlin, on 16th February 2017) and registered with data safety authorities. Study patients provided written informed consent to participate in the trial. Study registry: ClinicalTrials.gov, NCT03114306.

## Background

The local infiltration analgesia (LIA) and peripheral nerve block anaesthesia (PNBA) are techniques in the postoperative pain-management of patients undergoing major orthopaedic surgery such as total knee replacement.

PNBA – a regional anaesthesia procedure with/without catheters - is seen as an effective method in postoperative pain-management of endoprosthesis implantation [1]. The LIA was developed between 1998 and 2008 in Sydney, Australia [2, 3] aiming to reduce postoperative time to mobilisation and therefore leading to earlier discharge of patients into rehabilitation and, ultimately, enabling a faster return to preoperative physical activity levels [2]. In contrast to the PNBA, the LIA is not applied by the anaesthesiologist during the induction, but rather by the surgeon as part of the surgical procedure [3]. It is generally regarded as easy and therefore safe to administer [2, 3].

However, research-based discussion on the preferred method remains controversial [4, 5]. Some studies point to faster mobilisation with the LIA as a result of maintaining full functioning of the quadriceps-muscles [3]. This, in turn, is said to reduce time to hospital discharge, possible postoperative complications and therefore lead to faster rehabilitation [2]. Moreover, the LIA is seen as equally effective in treating postoperative pain as the PNBA [6] and is therefore increasingly accepted as a valid alternative [1, 7]. Nevertheless, there is evidence suggesting more significant postoperative pain on exertion in LIA patients when compared to PNBA [8]. The application of the PNBA is often considerably more time consuming [9] and requires specialised technical skills of the anaesthesiologist [2, 10] that are not required for the LIA. This can lengthen mean anaesthesia induction time significantly [10], which in turn may have an effect on overall anaesthesiological case costs.

Due to this ambivalent data, there is a rising interest in economic analyses complementing clinical and more patient-orientated aspects of determining which procedure is preferable [11]. Previous studies looking at financial aspects in perioperative pain management of total knee arthroplasty found that the implementation of the LIA lead to savings for hospitals [12], most prominently in material costs [13]. An assessment and comparison of the perioperative costs of LIA and PNBA has not been available to date. The aim of this study was to conduct a financial analysis of the immediate perioperative process and thus enable an economic evaluation of the LIA and the PNBA in addition to, and based on, Kastelik et al. [10].

## Methods

The study is a secondary analysis of a randomised controlled trial (RCT) published by Kastelik et al. (study registry: ClinicalTrials.gov, NCT03114306) [10]. The background to the trial, methods and baseline characteristics of the randomized patients have been previously reported. Its objective was to evaluate the two analgesic methods in postoperative pain management for patients receiving total knee arthroplasty (TKA). Forty Patients receiving primary TKA under general anaesthesia were included between April and August 2017 and randomised 1:1 (20 LIA vs. 20 PNBA). Exclusion criteria were heart or liver insufficiency, evidence of diabetic polyneuropathy, severe obesity, chronic opioid therapy for more than 3 months before scheduled surgery and allergy to local anaesthetics. Patients either received a total intravenous anaesthesia (TIVA) with propofol or a balanced anaesthesia with sevoflurane. The primary endpoint for Kastelik et al. was time taken to postoperative mobilisation (walking in the ward), which was achieved in both study arms on the first postoperative day (LIA 24.0 h versus PNBA 27.1 h, 95% confidence interval of − 9.6-3.3 h) [10].

The main subject of the secondary analysis at hand was the difference in overall case costs between LIA and PNBA procedures.

All patients received postoperative analgesia following a standardised protocol of opioids (tilidine/naloxone retard), dipyrone and non-steroidal anti-inflammatory drugs (NSAIDs, i.e. ibuprofen). For additional analgesic treatment in case of acute pain scores on the numeric rating scale (NRS) > 6, patients received 10 mg of oral morphine.

During the LIA procedure the surgeon injects 150 ml Ropivacain 0.2% into the periarticular structures, thereby blocking the sensitive nerve endings of the knee. The targeted structures are the subcutaneous and periarticular soft tissues as well as the joint capsule. LIA is often administered during the orthopaedic implantation of endoprosthesis, immediately before suture [10].

For PNBA, ultrasound-guided sub-gluteal block of the sciatic nerve as well as the adductor canal block with anaesthesia of the saphenous nerve were performed in the primary study [10]. Both nerve block procedures were administered just before induction of general anaesthesia [10]. In the calculations we considered the time taken to administer the PNBA as part of the anaesthesiological case costs (see below). For the blockage of the sciatic nerve 20 ml Ropivacain 0.75% were injected. The adductorial compartment was injected with 20 ml Prilocain 1% and a catheter was inserted for postoperative analgesia [10] via a patient controlled analgesia (PCA) system. Service life of the PCA was between 2 and 4 days (mean: 3.1 days). Patients with PNBA had regular visits by a pain nurse postoperatively.

### Calculation of costs

To calculate the overall case costs for each procedure we summed up the surgical case costs, anaesthesiological case costs, postoperative opioid requirements, material costs and costs of catheter review visits (only in PNBA procedure). The specification for the times used for the calculations was taken from Bauer et al. on perioperative process times and indicators [14] and is specified in brackets.

Surgical case costs were made up of the price per minute for personnel (doctor and surgical nurse), operational infrastructure and material (33.77€) and were multiplied by the incision to suture time (O8-O10 [14]). The execution of the LIA was part of the incision to suture time as it was administered by the surgeon during the operation.

Anaesthesiological case costs consisted of the price per minute for personnel (doctor and anaesthesiology nurse), infrastructure and material (10.28€) and were multiplied by the total time of anaesthesia (beginning of the anaesthesiologist’s presence with the patient until end of patient monitoring by the anaesthesiologist in the operating room (OR) or similar (A5-A9) [14]).

Material costs (Tables 1 and 2) were extracted from the surgical and anesthesiological case costs and examined separately for better comparison.

**Table 1:** Mean, standard deviation and *p*-values of incision to suture time and anaesthesia induction time

	LIA ***n*** = 20	PNBA ***n*** = 20	***P***
Incision to suture time (O8-O10 [14] in minutes)	87.0 ± 14.0	80.0 ± 16.0	0.076
Anaesthesia induction time (min) [10]	10.0 ± 3.0	35.0 ± 26.0	< 0.001

Opioid costs for the treatment of acute postoperative pain included the individual and average dosages of tilidine/naloxone retard and oral morphine 10 mg tablets [10]. The prices for 100 mg tilidine/naloxone retard was 1.40€, 10 mg morphine were 0.61€ based on internal pharmacy prices.

Catheter review-visits for patients receiving the PNBA were calculated with 6.25€ per visit based on the assumption of a before-tax income of 25€/h of an anaesthesiological nurse and an average time per visit of 15 min.

Total costs were obtained by the summation of the above variables. All costs and prices were taken from the expenses of the Charité–Universitätsmedizin Berlin at the time the study was conducted.

### Statistical methods

The statistical analysis of our data was conducted in IBM SPSS 25. Descriptive data was summarised by mean and standard deviation or median depending on statistical distribution. The t-test for independent samples was used to test for statistical significance of parametric data. Data for opioid requirement for postoperative acute pain as well as for induction costs were analysed using the Mann-Whitney-U-Test. For all statistical analysis a 5% two-sided alpha level was applied. A case number analysis was not conducted for this secondary end-point as this was a descriptive non-confirmatory supporting analysis. The statistical graphic was originally produced with SPSS 25 revised as vector graphics using Affinity Designer 1.8.6. Images were designed to be accessible for people with anomalous trichromacy, dichromacy and monochromacy.

## Results

Mean surgical case costs within the OR for the LIA procedure were 2933.81€ (SD ± 463.94€). For the PNBA mean costs were 2690.67€ (SD ± 532.67€). There was no statistically significant difference between the two procedures (*p* = 0.132).

Mean anaesthesiological case costs for the LIA were 1370.26€ (SD ± 209.54€) and 1542.45€ (SD ± 313.12€) for the PNBA. This difference was statistically significant (*p* = 0.048).

Table 1 shows the time differences in surgical and anaesthesiological inputs.

Material costs for the LIA were fixed within our hospital and amounted to 4.18€/patient and 94.64€/patient for the PNBA, respectively. Prices of the individual items are summarised in Tables 2 and 3. These are fixed costs for the respective procedure.

**Table 2:** Material costs local infiltration analgesia (LIA)

Material	Price €/Unit	Units/Patient
Ropivacain 0.2% 200 ml/bag	3.63	1
Injection needles	0.55	1
Total	4.18

**Table 3:** Material costs peripheral nerve block analgesia (PNBA)

Material	Prices €/Unit	Units/Patient
Administration Set	21.09	1
Sono-Block needle	11.38	1
Sterile ultrasound cover	3.38	1
Ultrasound gel, single use	0.95	1
Sterile gloves	1.10	1
Ropivacaine 0,2% 200 ml/bag	3.63	1
Ropivacaine 7,5 mg/ml, 10 ml	1.02	2
Lidocain 1%, 10 ml	0.21	1
Prilocain 1% 20 ml	1.62€	1
Surgical gown	3.37	1
Set for regional anaesthesia (Saphenus-Catheter)	46.89	1
Total	94.64	

There was no statistically significant difference in the cumulative use of postoperative opioids. Mean costs of cumulative postoperative tilidine/naloxone per patient were 19.01€ (SD ± 2.44€) for LIA and 17.22€ (SD ± 4.85€) for PNBA (*p* = 0.602). Mean costs of cumulative postoperative oral morphine per patient were 1.46€ (SD ± 2.08€) for LIA and 1.59€ for PNBA (SD ± 2.08€) (*p* = 0.646).

Mean costs for catheter review-visits amounted to 21.56€ per patient (SD ± 4.74€) and were added to the PNBA-group only, as outlined above.

**Fig. 1:**
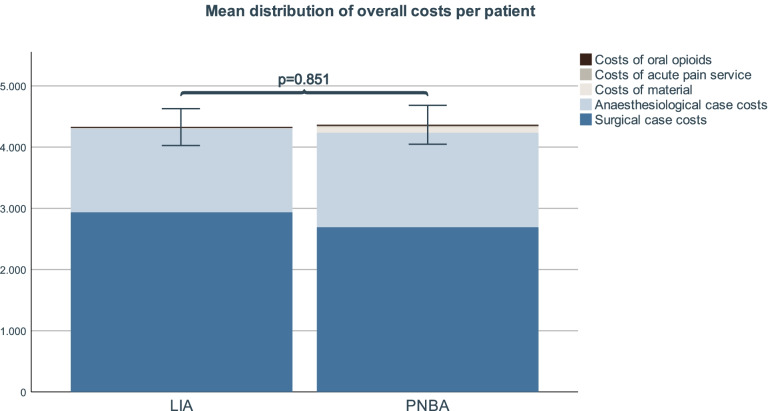
Stacked bar charts display mean distribution of separated costs per patient by study group. Error bars indicate 95% confidence interval for mean distribution of overall costs per patient

Overall, the mean costs for the LIA procedure were 4328.72€ (SD ± 644.14€) per patient compared to 4368.12€ (SD ± 677.24€) for the PNBA procedure. This difference was not statistically significant (*p* = 0.851). Figure 1 presents an overview of the individual positions of our calculations.

The authors of the primary study reported no significant postoperative complications that may have had an impact on costs with either procedure and there was no significant difference in total hospital length of stay between the two groups (*p* = .758) [10]. There was no data obtained on length of stay in the recovery room.

## Discussion

Our analysis shows that there seems to be no statistically significant difference in immediate perioperative costs between the two analgesic procedures in our hospital. Indeed, both concepts seem to provide fast-track mobilisation and allowed early in-hospital recovery after surgery and ambulation maintaining high patient comfort with comparable costs (patient-related outcome measure).

Although the results show that the anaesthesiological case costs were significantly lower for the LIA procedure, this was primarily due to the prolonged anaesthesia induction time for the PNBA [10] and will depend largely on institutional structure and professional experience of personnel in different hospitals. For example, PNBA could be administered while the preceding operation and therefore anaesthesia is still under way.

Kastelik et al. reported a longer mean incision to suture time in the LIA group [10] which, although not statistically significant, may explain the slight and equally insignificant difference in surgical case costs within the OR. The study, however, was not aimed towards this secondary endpoint and might thus have been underpowered in this regard. Nevertheless, it might be worth noting that performing LIA is not uniformly standardized especially when infiltrating the posterior capsule. In this context the surgeon’s experience with LIA is of interest in exploiting full cost saving potential.

Material costs were considerably lower in the LIA group, which was consistent with existing research [13]. Other than our focus on the perioperative processing costs past studies analysing financial aspects of different analgesic procedures in treatment of TKA put a larger focus on hospitalisation time [7, 12], required material [13] or overall financial burden for society and healthcare system [15]. Existing studies with an emphasis on cost efficiency in a perioperative setting, in turn, were aimed more towards a comparison between regional and systemic [16] rather than local analgesia such as LIA. Alongside considerable material costs, they often also stressed the importance of increased personnel expenditure required for application and postoperative care for regional anaesthesia procedures [8, 17], which was also an important factor in our analysis. In the existing research the regional anaesthesia performed better from an economical “overall” point of view [8, 9]. This is mainly explained by shorter stays in the recovery room and optimized operational processes [17]. However, an advantage of regional over local analgesia could not be found in our study. Other comprehensive data on the economic comparison between procedures of regional and local anaesthesia as well as on our focus of operational times and associated costs is not available yet.

One objective of the development of the LIA was to reduce risk of postoperative complications such as venous thrombosis or nosocomial infection [2] achieved by faster mobilisation, shorter overall hospitalisation, and forgoing invasive catheters as required with the PNBA. Financial savings and healthcare benefits that may thus be achieved in the long run by widespread implementation of the LIA were not subject of our analysis and remain to be examined. However, existing research suggests that the LIA may have the potential to improve quality of life and reduce long-term public sector spending on healthcare when compared to other analgesic methods [15].

Mobilisation to stand was successfully achieved in the recovery room in 90% of LIA and 95% of PNBA patients in the primary study [10]. This may give an indication that other block procedures would not necessarily lead to a superior outcome regarding time to mobilisation or hospital total length of stay and therefore relating costs.

Limitations of our work may include the restricted transferability of cost structures of a university hospital like the Charité to other institutions on a national and international level. The surgical case costs as the largest cost factor may differ depending on the surgeon’s experience in the handling of the LIA and the overall procedure of TKA. Another limitation may be the relatively small sample size (*N* = 40), which was powered for the primary endpoint of mobilization. Thus it may be insufficient to draw conclusive evidence towards economical differences between the two procedures without further research. However, due to the randomization in the study design risk of confounding bias stays relatively small. Further research should expand on these issues and potentially include other postoperative costs such as physical rehabilitation and overall hospital length of stay.

## Conclusions

This study expands on the financial evaluation for determining a preferred procedure in perioperative pain management for patients receiving TKA. Although we were able to describe in detail lower anaesthesiological case costs with the LIA, there was no difference found between the overall case costs for LIA and PNBA. Therefore, based on our analysis we can conclude that the organisational structure and process optimization are the key to reduce costs for either procedure.

It should be noted that the determination of an analgesic procedure for patients receiving knee replacement surgery should always rely primarily on medical indications and should therefore be chosen by carefully evaluating individual risk factors and benefits for each individual patient. Economic factors should only be seen as secondary arguments for the individual but can play a considerable role in the effective allocation of resources and organisational planning.

## Data Availability

The datasets generated and/or analyzed during the current study are not publicly available due to German data protection regulations regarding the submission of study data to a public data depot. Upon reasonable request data can be made available in an anonymized format by the corresponding author.

## Abbreviations

LIA: Local Infiltration Analgesia
PNBA: Peripheral Nerve Block Anaesthesia
OR: Operating Room
TKA: Total Knee Arthroplasty
TIVA: Total Intravenous Anaesthesia
NSAID: Non-Steroidal Anti-Inflammatory Drug
NRS: Numeric Rating Scale
PCA: Patient Controlled Analgesia
OR: Operating Room
